# Macrophage depletion in inflamed rat knees prevents the activation of synovial mesenchymal stem cells by weakening Nampt and Spp1 signaling

**DOI:** 10.1186/s41232-024-00361-2

**Published:** 2024-11-20

**Authors:** Hayato Kodama, Kentaro Endo, Ichiro Sekiya

**Affiliations:** 1 Center for Stem Cell and Regenerative Medicine, Institute of Science Tokyo, 1-5-45 Yushima, Bunkyo-ku, Tokyo, 113-8510, Japan; 2 Center for Stem Cell and Regenerative Medicine, Tokyo Medical and Dental University (TMDU), 1-5-45 Yushima, Bunkyo-Ku, Tokyo, 113-8510 Japan

**Keywords:** Macrophage, Mesenchymal stem cell, Clodronate liposome, Synovium, Single-cell RNA sequencing

## Abstract

**Background:**

Macrophages and mesenchymal stem cells (MSCs) engage in crucial interplay during inflammation and have significant roles in tissue regeneration. Synovial MSCs, as key players in joint regeneration, are known to proliferate together with macrophages in synovitis. However, the crosstalk between synovial MSCs and macrophages remains unclear. In this study, we investigated changes in the activation of synovial MSCs in inflamed rat knees following selective depletion of macrophages with clodronate liposomes.

**Methods:**

Acute inflammation was induced in rat knee joints by injection of carrageenan (day 0). Clodronate liposomes were administered intra-articularly on days 1 and 4 to deplete macrophages, with empty liposomes as a control. Knee joints were collected on day 7 for evaluation by histology, flow cytometry, and colony-forming assays. Concurrently, synovial MSCs were cultured and subjected to proliferation assays, flow cytometry, and chondrogenesis assessments. We also analyzed their crosstalk using single-cell RNA sequencing (scRNA-seq).

**Results:**

Clodronate liposome treatment significantly reduced CD68-positive macrophage numbers and suppressed synovitis. Immunohistochemistry and flow cytometry showed decreased expression of CD68 (a macrophage marker) and CD44 and CD271 (MSC markers) in the clodronate group, while CD73 expression remained unchanged. The number of colony-forming cells per 1000 nucleated cells and per gram of synovium was significantly lower in the clodronate group than in the control group. Cultured synovial MSCs from both groups showed comparable proliferation, surface antigen expression, and chondrogenic capacity. scRNA-seq identified seven distinct synovial fibroblast (SF) subsets, with a notable decrease in the Mki67^+^ SF subset, corresponding to synovial MSCs, in the clodronate group. Clodronate treatment downregulated genes related to extracellular matrix organization and anabolic pathways in Mki67^+^ SF. Cell–cell communication analysis revealed diminished Nampt and Spp1 signaling interaction between macrophages and Mki67^+^ SF and diminished Ccl7, Spp1, and Csf1 signaling interaction between Mki67^+^ SF and macrophages in the clodronate group. Spp1 and Nampt promoted the proliferation and/or chondrogenesis of synovial MSCs.

**Conclusions:**

Macrophage depletion with clodronate liposomes suppressed synovitis and reduced the number and activity of synovial MSCs, highlighting the significance of macrophage-derived Nampt and Spp1 signals in synovial MSC activation. These findings offer potential therapeutic strategies to promote joint tissue regeneration by enhancing beneficial signals between macrophages and synovial MSCs.

**Supplementary Information:**

The online version contains supplementary material available at 10.1186/s41232-024-00361-2.

## Background

The synovium is a thin membrane that plays a crucial role in maintaining normal joint function. Synovial tissue is structured into lining and sublining layers and consists primarily of synovial fibroblasts (SFs) and macrophages [1]. When a joint is damaged, these cells act in a coordinated manner to facilitate tissue repair [2] by coordinating with synovial mesenchymal stem cells (MSCs), which are distributed throughout the synovium and function as key players for endogenous tissue regeneration [3]. Synovial MSCs have great self-renewal, chondrogenic, and anti-inflammatory abilities [4] and express CD44 and CD73 in the synovial lining and sublining layers, respectively [5]. These cells are normally in a quiescent state, but actively proliferate when articular cartilage is damaged to ensure cartilage repair [6].

One signal that elicits this proliferation response is inflammation, a process partially regulated by the activities of macrophages, which also play important roles in the regeneration of various tissues [7]. Macrophages display diverse phenotypes that are broadly classified into inflammatory M1 and regulatory M2 phenotypes [8]. M1 macrophages are positive for CD80/CD86 expression and mediate an inflammatory response by producing proinflammatory cytokines and nitric oxide synthase [9]. By contrast, M2 macrophages express CD163/CD206 and regulate inflammation and tissue remodeling through the secretion of anti-inflammatory cytokines, such as interleukin-10 (IL-10) and transforming growth factor-β (TGF-β) [10, 11]. Previous studies have demonstrated that the polarization of macrophages is reversible and reciprocal, with the M1/M2 ratio adjusting according to the surrounding environment [8, 12, 13]. During acute synovitis, the number of macrophages increases considerably. This increase is caused by the self-renewal of synovium-resident macrophages in the sublining layer and an influx of monocyte-derived macrophages from blood vessels [14]. The resolution of inflammation requires that macrophages and synovial MSCs participate in crosstalk to enable cooperative switching of their respective phenotypes to a pro-reparative state [15].

This crosstalk between MSCs and macrophages has recently inspired great research interest. Once inflammation or damage occurs, macrophages secrete pro-inflammatory factors, such as IL-1β and TNF-α. MSCs receive these signals and shift toward more immunomodulatory and tissue reparative phenotypes, and in turn communicate back to macrophages by secreting various signals, including cytokines, growth factors, and exosomes [16]. For example, in skeletal muscle, macrophages and muscle stem cells are reported to migrate to a site of injury and communicate with each other to induce muscle stem cell proliferation and differentiation [17]. Although we have previously reported that the numbers of synovial MSCs and macrophages increase intensely during acute synovitis, the crosstalk between these synovial cells has never been investigated [18]. Unraveling their crosstalk mechanisms will aid in controlling and optimizing the process of joint tissue regeneration by synovial MSCs and macrophages.

In the present study, we selectively depleted macrophages in inflamed rat knee joints using clodronate liposomes [19, 20] to investigate changes in synovial MSC activation, including MSC numbers and function. We also analyzed their crosstalk using single-cell RNA sequencing (scRNA-seq).

## Methods

### Animals

**Fig. 1:**
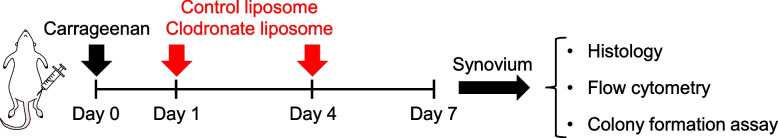
Experimental scheme. Carrageenan was injected into rat knees to induce inflammation (day 0). On days 1 and 4, control liposomes or clodronate liposomes were injected into the inflamed rat knees to deplete macrophages. On day 7, the knee joints were evaluated by histology, flow cytometry, and colony-formation assays

Ten-week-old wild-type male Lewis rats (Sankyo Labo Service Corporation, Tokyo, Japan) were used for the experiments. All animal care and experiments were conducted in accordance with the institutional guidelines of the Animal Committee of Tokyo Medical and Dental University (permission number: A2021-267A) and the ARRIVE guidelines. To induce acute inflammation, 1% carrageenan (50 µL) was injected into the knee joint on day 0. Subsequently, on days 1 and 4, the rats received an intra-articular injection of clodronate liposomes or empty control liposomes (0.1 mg/50 µL; HYGIEIA BIOSCIENCE, Osaka, Japan). Rats were euthanized by carbon dioxide inhalation and the knee joints were collected at day 7 for histological evaluation, flow cytometry, and colony-formation assays (Fig. 1). Intact knees without inflammation (no carrageenan administration) also received clodronate or control liposome injections in the same way.

### Histology

The collected tissues were fixed at room temperature in 10% formalin neutral buffer solution (Wako, Osaka, Japan) for 7 days, decalcified in 20% ethylenediaminetetraacetic acid (EDTA) for 21 days, and then embedded in paraffin. Sagittal Sects. (5 µm thickness) were stained with hematoxylin/eosin (HE) and the degree of synovitis was scored using the Krenn score [21]. All images were captured using a microscope (BZ-X700, KEYENCE, Osaka, Japan).

### Immunohistochemistry

The sections were incubated in 10 mM Tris containing 1 mM EDTA (pH 9.0) at 60 °C for 16 h for antigen retrieval. The slides were then incubated in methanol containing 0.3% H_2_O_2_ for 30 min, followed by washing with Tris-buffered saline containing 0.1% Tween-20 (TBS-T). After blocking with Blocking One Histo (NACALAI TESQUE, Kyoto, Japan), the sections were incubated overnight at 4 °C with antibody against CD68 (1:200; Novus Biologicals, Littleton, CO, USA), CD80 (1:200; Bioss Antibodies, Woburn, MA, USA), CD206 (1:1000; Abcam, Cambridge, UK), CD44 (1:200; Proteintech, Rosemont, IL, USA), CD73 (1:200; R&D Systems, Minneapolis, MN, USA), and CD271 (1:500; Millipore, Billerica, MA, USA). After three washes with TBS-T, diaminobenzidine (DAB) solution (Dako, Glostrup, Denmark) was applied for 5 min, and the cells were counterstained with hematoxylin. We quantified the DAB-positive areas using the Color Deconvolution plugin for Fiji/Image J (National Institute of Health, Bethesda, MD, USA) in two fields of view (150 × 150 µm). After unmixing the hematoxylin and DAB colors, we determined the DAB-positive area using thresholding binary images. All images were acquired under the same light settings.

### Flow cytometry

We minced and digested synovial tissues with 0.4 mg/mL Liberase (Roche Diagnostics, Mannheim, Germany) and 0.1 mg/mL DNase I (Sigma-Aldrich, St. Louis, MO, USA) at 37 °C for 3–4 h. Residual erythrocytes were lysed in ACK Lysing Buffer (Thermo Fisher Scientific, Waltham, MA, USA) and the cells were passed through a 70 µm strainer to remove debris (Greiner Bio-One GmbH, Frickenhausen, Germany). After staining with Ghost Dye Violet 510 for dead cells (Tonbo Biosciences, CA, USA), the cells were permeabilized with Fixation and Permeabilization solution (BD, San Jose, CA, USA) and suspended in FACS buffer (PBS with 2% fetal bovine serum (FBS) and 5 mM EDTA). Cells were then stained with CD44-PE (R&D Systems), CD68-FITC (BIO-RAD, Hercules, CA, USA), CD73-PE-Cy7 (Bioss), CD80-BV421 (BD), CD86-Alexa647 (BIO-RAD), CD90-BV421 (BD), CD105-APC (Novus), CD206-PE (Bioss), and CD271-Alexa488 (BD) antibodies at 4 °C for 1 h. The fluorescence signals were detected using a FACS Melody instrument (BD). The proportion of antigen-positive cells was evaluated using FlowJo software (BD).

### Colony formation assay

Synovial tissues were digested in the same manner described for flow cytometry. After cell counting, 1000 cells were plated into a 145 cm^2^ dish (Thermo Fisher Scientific) and cultured in growth medium consisting of α-modified essential medium (α-MEM; Thermo Fisher Scientific), 10% FBS (Thermo Fisher Scientific), and 1% antibiotic–antimycotic (Thermo Fisher Scientific) for 2 weeks to form colonies. Cells were then fixed in 10% neutral buffered formalin and stained with 0.5% crystal violet. The colonies were counted manually, ignoring colonies less than 5 mm in diameter. The number of colony-forming cells per gram of synovium was calculated using the cell number per gram of synovium and the colony number per dish.

### Rat synovial MSC isolation

Synovial cells obtained by synovium digestion were seeded in a 145 cm^2^ dish (Thermo Fisher Scientific) at a density of 500 cells/cm^2^ and cultured in growth medium. Confluent cells were detached with 0.25% trypsin and 1 mM EDTA (Thermo Fisher Scientific) and cryopreserved in FBS supplemented with 5% dimethyl sulfoxide (Wako, Tokyo, Japan) for future use. Passage 1 cells were used for the experiment.

### Proliferation assays

Rat synovial MSCs were plated in 96-well plates at a density of 500 cells/cm^2^. After 2, 4, and 6 days of cultivation, the number of cells in each well was evaluated using a Cell Counting Kit-8 (Dojindo, Kumamoto, Japan) according to the manufacturer’s instructions. The optical density values at 450 nm were measured with a microplate reader (Tecan, Männedorf, Switzerland). The cells were then fixed with 10% neutral-buffered formalin and stained with 0.5% crystal violet. The effects of Spp1 and Nampt were evaluated by stimulating rat synovial MSCs with 0–1000 ng/mL recombinant human Spp1 and Nampt (both from Pepro Tech, Rocky Hill, NJ, USA) in the presence of 2% FBS. After 7 days of culture, the numbers of cells were counted using a Cell Counting Kit-8.

### Chondrogenesis

For chondrogenesis, 2.5 × 10^5^ cells were centrifuged at 580 × g for 10 min in 15 mL tubes (Thermo Fisher Scientific). The cell pellets were incubated in high glucose Dulbecco’s Modified Eagle Medium (Thermo Fisher Scientific), 1% insulin-transferrin-selenium (Corning, NY, USA), 50 µg/mL ascorbate-2-phosphate, 40 µg/mL l-proline (Sigma-Aldrich), 100 nM dexamethasone, 100 µg/mL pyruvate (Sigma-Aldrich), 1% antibiotic–antimycotic, 10 ng/mL human transforming growth factor-β3 (Miltenyi Biotec Japan, Tokyo, Japan), and 500 ng/mL human bone morphogenetic protein 2 (Medtronic, Minneapolis, MN). After 3 weeks of cultivation, we performed Safranin O/Fast Green (Wako) staining, DNA and glycosaminoglycan (GAG) quantification, and quantitative real-time PCR (qPCR). The effects of Spp1 and Nampt were evaluated by treating rat synovial MSCs were treated with 1000 ng/mL of Nampt or Spp1 in the presence of 2% FBS for 7 days and then performing the chondrogenic assay.

### DNA and GAG quantification

Chondrogenic pellets were digested with 100 µg/mL papain (P3125; Sigma-Aldrich) at 65 °C for 16 h. The DNA content was measured using Hoechst 33,258 dye (H341; Dojindo). Fluorescence signals were measured with a microplate reader (Infinite M200; Tecan) at an excitation wavelength of 360 nm and an emission wavelength of 465 nm. Calf thymus DNA (D4522; Sigma-Aldrich) was used to generate a standard curve. The GAG content was measured using a Blyscan Kit (B1000; Biocolor, Westbury, NY, USA) according to the manufacturer’s instructions. The optical density at 656 nm was measured using a microplate reader. The total GAG content was normalized to the total DNA content (GAG/DNA). This experiment was performed on triplicate pellets.

### Quantitative real-time PCR

We extracted total RNA using the miRNeasy Mini Kit (217,004; QIAGEN, Venlo, Netherlands) according to the manufacturer’s instructions. Total RNA concentration was measured using a NanoDrop instrument (Thermo Fisher Scientific). We synthesized first-strand complementary DNA using a ReverTra Ace qPCR RT Master Mix with gDNA Remover (TOYOBO Corporation, Osaka, Japan). PCR was performed by real-time monitoring using the Thunderbird SYBR qPCR Mix (QPS-201; Toyobo) on a LightCycler 480 instrument II (Roche Diagnostics). The following primers were used: *β*-*actin*, 5′-GCAGGAGTACGATGAGTCCG-3′ (forward) and 5′-ACGCAGCTCAGTAACAGTCC-3′ (reverse); *SOX-9*, 5′-ATCTTCAAGGCGCTGCAA-3′ (forward) and 5′-CGGTGGACCCTGAGATTG-3′ (reverse); *Aggrecan*, 5′-TGGCTGCAGGACCAGACT-3′ (forward) and 5′-CGCCATAGGTCCTGACTCC-3′ (reverse); *COL1A1*, 5′-TCCTGCCGATGTCGCTATC-3′ (forward) and 5′-CCATGTAGGCTACGCTGTTCTTG-3′ (reverse); *COL2A1*, 5′-CTTTCCTCCGTCTACTGTCCA-3′ (forward) and 5′-GCCCTCATCTCCACATCATT-3′ (reverse); *COL10A1*, 5′-GGTCCACCAGGTCCACAA-3′ (forward) and 5′-TGGCTCCCAATACCTTCTC-3′ (reverse). The cycling conditions were 45 cycles at 95 °C for 15 s and 60 °C for 30 s. β-actin was used as an internal control.

### scRNA-seq analysis

Synovial tissues were digested as described for flow cytometry. After lysing erythrocytes with ACK Lysing Buffer, the digested cells were cryopreserved in Cellbanker 1Plus (Zenoaq, Fukushima, Japan). The scRNA-seq was performed by the Beijing Genomics Institute (BGI; Shenzhen, Guang-dong, China) using the DNBseq platform. Raw data were aligned to the rat reference genome (ensembl_102) using STAR aligner and processed in Cell Ranger v7.2.0. The processed data were analyzed using Seurat (R, v5.1.0). After filtering out low-quality data (> 10% mitochondrial reads), the remaining data were normalized to 10,000 reads multiplied by a scale factor. The data for the control and clodronate groups were integrated using the “FindIntegrationAnchors” algorithm. After linear dimensional reduction, cell clustering was performed at dims 1:20 and resolution 0.4 and visualized on a uniform manifold approximation and projection (UMAP) plot. We identified 20 distinct clusters (Supplementary Fig. 2) and annotated them based on the expression of the following marker genes: *Cd68* and *Cd80* for M1 macrophages; *Cd68* and *Cd206* for M2 macrophages; *Cd68* and *Mki67* for proliferating macrophages; *Cd3e* and *Cd3g* for T cells; *Ecn* and *Cdh5* for endothelial cells; *Acta2* and *Tagln* for smooth muscle cells; *Cd79a* and *Cd79b* for B cells; *Myod1* and *Mybpc1* for myoblasts; and *Sox10* and *Mpz* for Schwann cells (Supplementary Fig. 3). The remaining SFs were named mainly according to the expression levels of previously reported feature genes [22–24]. Differentially expressed genes (DEGs) between clusters and groups were defined by min.pct > 0.1 and log FC > 0.5. We used clusterProfiler (4.12.0) [25] for gene ontology (GO) analysis. Pseudotime trajectory analysis for seven distinct SF subsets was conducted using monocle3 (v1.3.1)[26] and slingshot (v2.12.0) [27]. Cell–cell communication was analyzed and visualized with CellChat (v2.1.2) [28].

### Statistical analysis

All statistical analyses were performed using GraphPad Prism 10 software (GraphPad Software, CA, USA). Student’s *t* test was used and a *P* value < 0.05 was considered statistically significant.

## Results

### Verification of the effect of clodronate liposome treatment in intact knees

We first verified the effect of clodronate liposomes in intact rat knees. In control liposome-injected knees, CD68 expression was observed mainly on the synovial surface and around blood vessels, whereas clodronate liposome–treated knees showed less expression at these sites (Supplementary Fig. 1A). The CD68-positive area was significantly decreased in the clodronate group, indicating that clodronate liposomes effectively eliminate macrophages (Supplementary Fig. 1B).

### Clodronate liposome treatment suppressed synovitis in carrageenan-injected knees

**Fig. 2:**
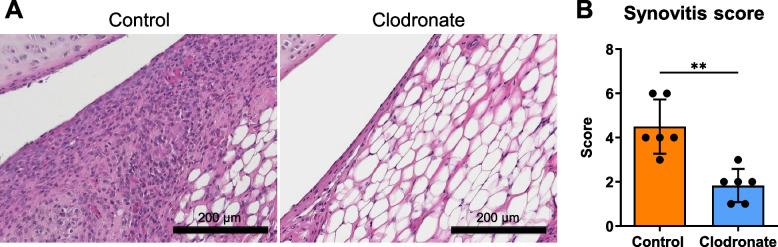
Histological evaluation of the rat knee synovium. **A** Representative images of synovium stained with hematoxylin and eosin. **B** Synovitis score. Data are presented as the mean ± SD of 6 knees. **p < 0.01

Carrageenan was injected into the rat knee joint to induce acute inflammation, followed by the injection of control and clodronate liposomes (Fig. 1). The control group showed severe thickening of the synovial surface layer and high cell density in the stroma. By contrast, the clodronate group showed 2–3 cell layers on the synovial surface and a lower stroma cell density than the control group. Krenn’s synovitis score was significantly lower in the clodronate group (Fig. 2).

### Clodronate liposome treatment decreased the number of macrophages and MSCs

**Fig. 3:**
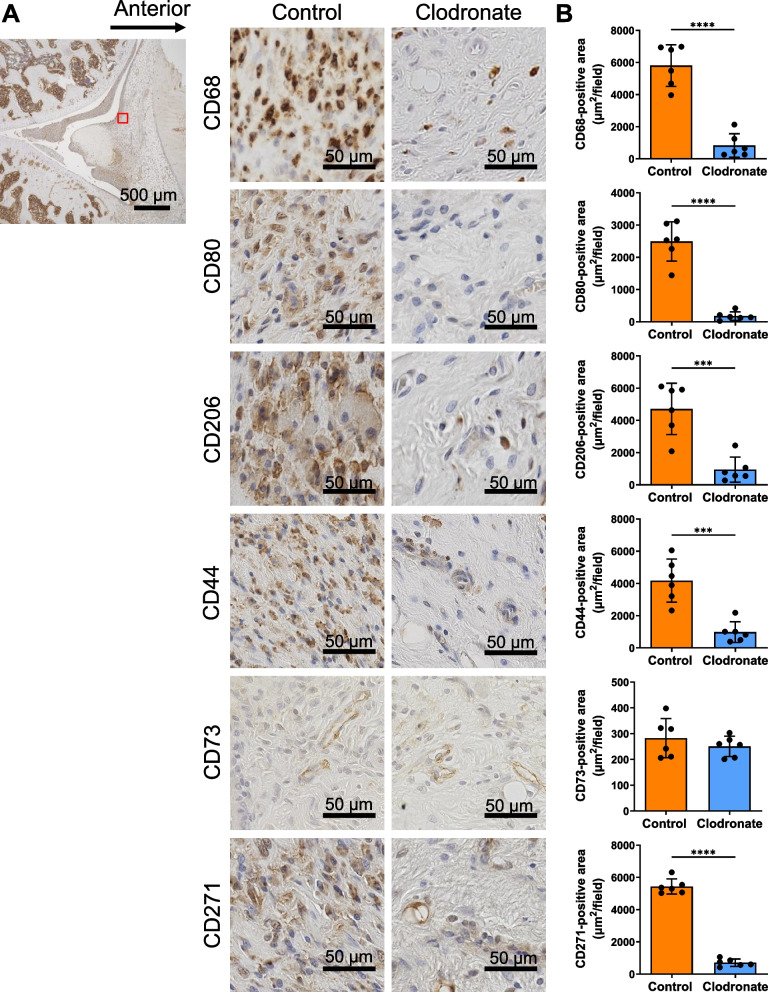
Immunohistochemistry of rat knee synovium. **A** Representative images of CD68, CD80, CD206, CD44, CD73, and CD271. **B** Quantification of the positive areas. Data are presented as the mean ± SD of 6 knees. ***p < 0.005 and ****p < 0.0005

We evaluated the synovial expression of macrophage (CD68, CD80, and CD206) and MSC markers (CD44, CD73, and CD271) by immunostaining. Although many CD68-, CD80-, and CD206-expressing cells were observed in the control group, their numbers were markedly decreased in the clodronate group (Fig. 3A). CD68-, CD80-, and CD206-positive areas were all significantly decreased in the clodronate group (Fig. 3B). The CD44- and CD271-positive areas were also significantly decreased in the clodronate group. However, no obvious differences were noted in CD73 expression between the two groups.

### Clodronate liposome treatment decreased the ratio of macrophages and MSCs

**Fig. 4:**
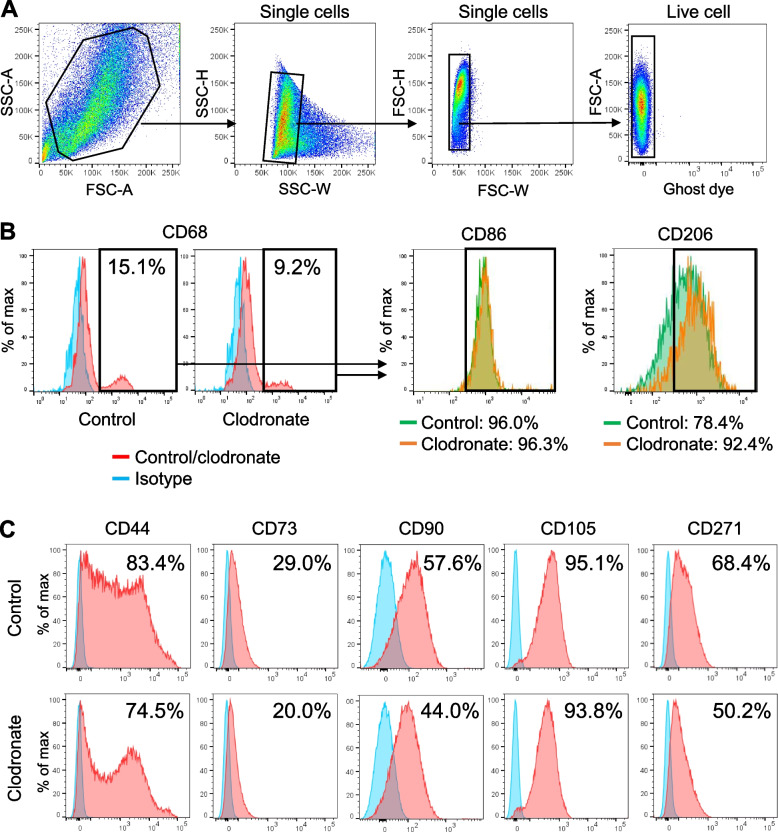
Flow cytometry of synovium-derived cells. **A** Gating strategy. Debris, doublets, and dead cells were excluded from the analysis. **B** Positive rate for macrophages markers. **C** Positive rate for MSC markers. The experiment was repeated twice and representative data are shown. *FSC* forward scatter, *SSC* side scatter

Flow cytometry of synovial tissue confirmed a lower percentage of CD68-positive cells in the clodronate group (Fig. 4 A and B). We assessed the changes in M1/M2 polarization by also evaluating the CD86 and CD206 expressions in CD68-positive cells. Although no difference was evident in the expression of CD86, the peak of the CD206 signal shifted to a higher intensity in the clodronate group than in the control group. The proportion of cells positive for the MSC markers CD44, CD73, CD90, and CD271 was lower in the clodronate group than in the control group (Fig. 4C). Both groups showed a comparable proportion of CD105-positive cells.

### Clodronate liposome treatment decreased the ratio and number of colony-forming cells

**Fig. 5:**
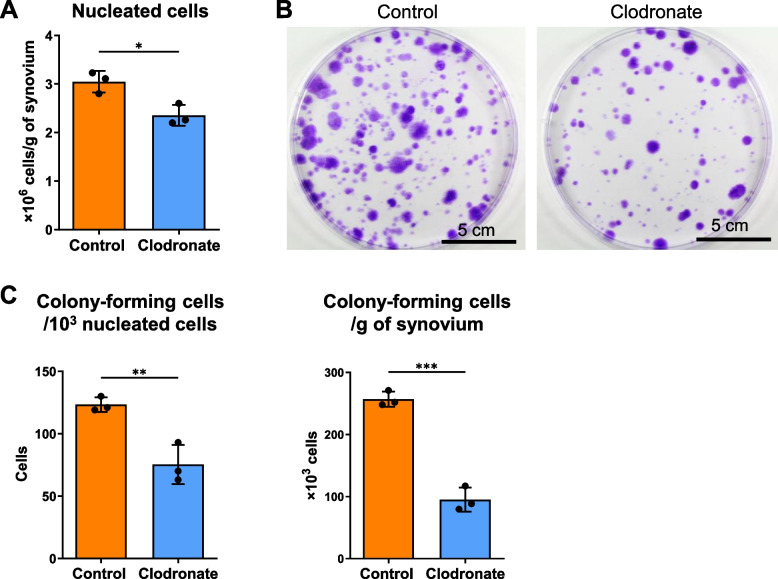
Numbers of colony-forming cells from synovium. **A** The number of nucleated cells per gram of synovium. Data are presented as the mean ± SD of three knees. **B** Representative images of cell colonies stained with crystal violet. **C** The numbers of colony-forming cells per dish and per gram of synovium. Data are presented as the mean ± SD of three replicate dishes. *p < 0.05, **p < 0.01, and ***p < 0.005

Counting of nucleated cells from rat synovia after Liberase digestion revealed a significant decrease in the number of nucleated cells per gram of synovium in the clodronate group (Fig. 5A). Colony formation assays performed to assess the ratio and number of colony-forming cells also revealed a significant decrease in the number of colony-forming cells per 1000 nucleated cells in the clodronate group (Fig. 5 B and C). The resulting calculated number of colony-forming cells per gram of synovium was considerably lower in the clodronate group than in the control group.

### Cultured MSCs showed equivalent proliferative potential and antigen expression

**Fig. 6:**
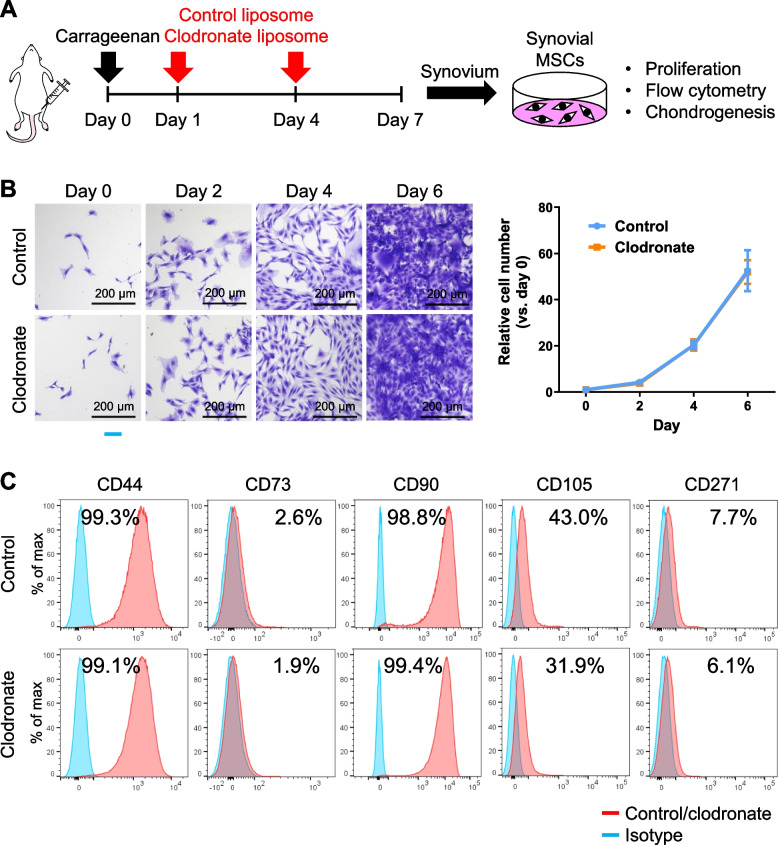
Proliferative ability and surface marker expression of isolated synovial MSCs. **A** Experimental scheme. Synovial MSCs were isolated from the synovium of rat knees injected with control or clodronate liposomes. Nucleated synovial cells from 6 knees of 3 rats from each group were pooled to obtain synovial MSCs, and P1 cells were used for the analyses. **B** Proliferation assay. Cell morphology and relative cell numbers on days 0, 2, 4, and 6. Data are presented as the mean ± SD of 6 replicate wells. **C** Expression of surface markers. The experiment was repeated twice and representative data are shown

We cultured synovial MSCs from rats injected with control liposomes and clodronate liposomes and performed proliferation assays, flow cytometry, and chondrogenic differentiation assays (Fig. 6A). We observed no differences in cell morphology and proliferative potential between the two groups (Fig. 6B). Surface antigen expression profiles were also comparable for both groups (Fig. 6C).

### MSCs cultured from the clodronate group formed larger cartilage pellets

**Fig. 7:**
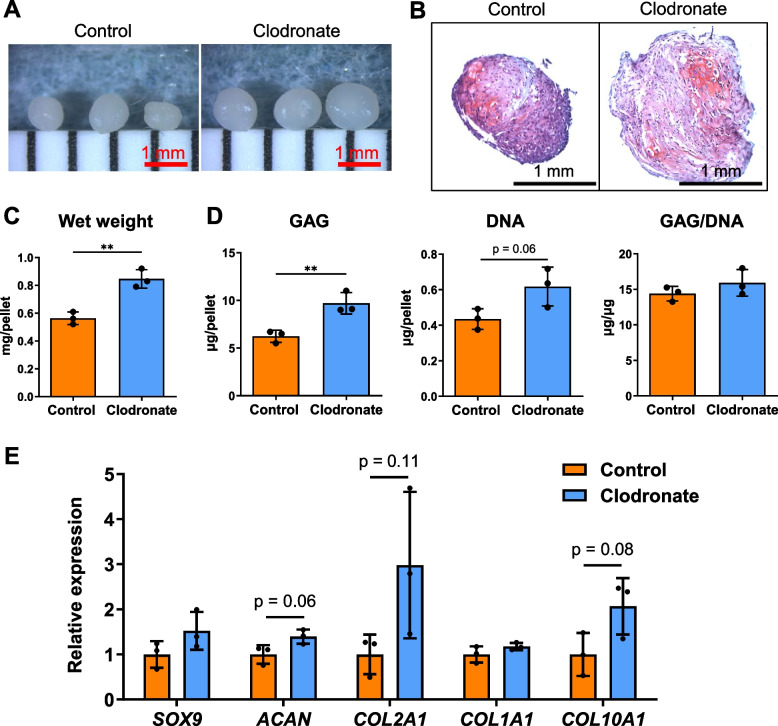
Chondrogenic ability of P1 synovial MSCs. **A** Gross appearance of chondrogenic pellets. **B** Representative images after safranin O/fast green staining. **C** Pellet wet weight. Data are presented as the mean ± SD of three replicate pellets. **D** Quantification of glycosaminoglycan (GAG) and DNA. The GAG content was standardized to DNA content (GAG/DNA). Data are presented as the mean ± SD of three replicate pellets. **E** Quantification of chondrogenic genes. Data are presented as the mean ± SD of three replicate pellets. **p < 0.01

Under gross observation, the chondrogenic pellets were larger in size in the clodronate group than in the control group (Fig. 7A). The pellets in both groups stained positive for safranin O staining (Fig. 7B). Pellet wet weight and GAG and DNA contents were increased in the clodronate group, but no significant difference was observed for the GAG/DNA ratio (Fig. 7 C and D). The mRNA expression of *ACAN*, *COL2A1*, and *COL10A1* tended to be elevated in the clodronate group (Fig. 7E).

### scRNA-seq analysis revealed seven distinct SF subsets and their inferred trajectories

**Fig. 8:**
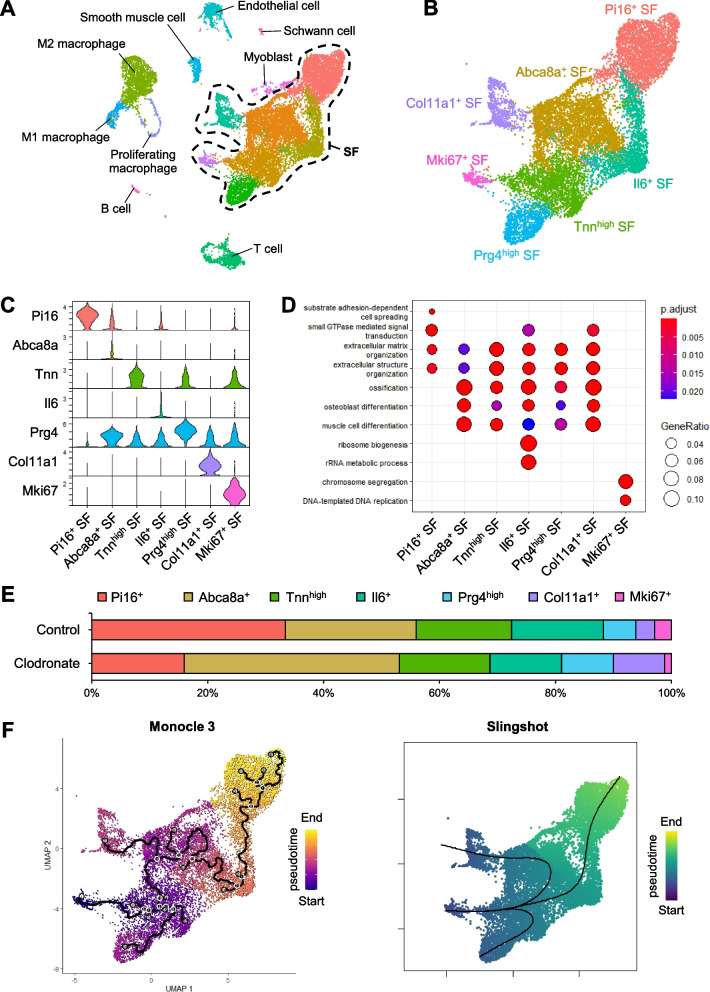
Single-cell RNA sequencing of synovial cells from the control and clodronate groups. **A** Uniform manifold approximation and projection (UMAP) plot showing synovial cell clusters after the cell type annotation. **B** UMAP plot showing SF subsets. **C** Violin plot showing the expression pattern of marker genes in each SF subset. **D** Dotplot showing gene ontology analysis of differentially expressed genes that are highly expressed in each SF cluster. **E** Proportional breakdown of each SF subset in each condition. **F** Pseudotime trajectory analysis of SF subsets using monocle3 and slingshot. The black line shows the trajectory plot of cell fates

We analyzed the crosstalk between synovial MSCs and macrophages using scRNA-seq. We analyzed 8852 control group and 10,248 clodronate group cells and identified 20 distinct clusters (Supplementary Fig. 2). After integrating the data, we classified the cells into 16 distinct cell types (M1 macrophage, M2 macrophage, proliferating macrophage, endothelial cell, smooth muscle cell, T cell, B cell, myoblast, Schwann cell, and seven SF subsets) based on the expression of marker genes (Fig. 8A and Supplementary Fig. 3). The proportion of macrophages was similar in both groups (Supplementary Fig. 4A). Regarding the distribution of each type of macrophage, the clodronate group had a decreased percentage of M1 macrophages and an increased percentage of proliferating macrophages (Supplementary Fig. 4B). The seven SF subsets were named Pi16^+^ SF, Abca8a^+^ SF, Tnn^high^ SF, Il6^+^ SF, Prg4^high^ SF, Col11a1^+^ SF, and Mki67^+^ SF based on the expression levels of *Pi16*, *Abca8a*, *Tnn*, *Il6*, *Prg4*, *Col11a1*, and *Mki67* [22–24] (Fig. 8 B and C). The SF subsets showed clear segregation from each other on a heatmap drawn using the top 10 DEGs (Supplementary Fig. 5). GO analysis of biological processes for DEGs that were highly expressed in each cluster revealed an enrichment of genes related to ECM organization in the SF subsets other than Mki67^+^ SF (Fig. 8D). DNA replication- and chromosome segregation-related terms were identified only in the Mki67^+^ SF subset. A proportional breakdown of each subset in each condition is shown in Fig. 8E. The proportion of Mki67^+^ SF among all SF subsets was lower in the clodronate group (0.85%) than in the control group (2.15%). Conversely, the proportion of Col11a1^+^ SF was higher in the clodronate group.

We identified an inferred developmental origin of SFs by performing two unsupervised pseudotime trajectory analyses: monocle3 and slingshot [26, 27]. Both algorithms depicted a cell lineage starting at Mki67^+^ SF and ending at Prg4^high^ SF, Col11a1^+^ SF, and Pi16^+^ SF via Tnn^high^ SF, Abca8a^+^ SF, and IL6^+^ SF, respectively (Fig. 8F).

### Clodronate liposome treatment altered communications between Mki67^+^ SF and macrophages

**Fig. 9:**
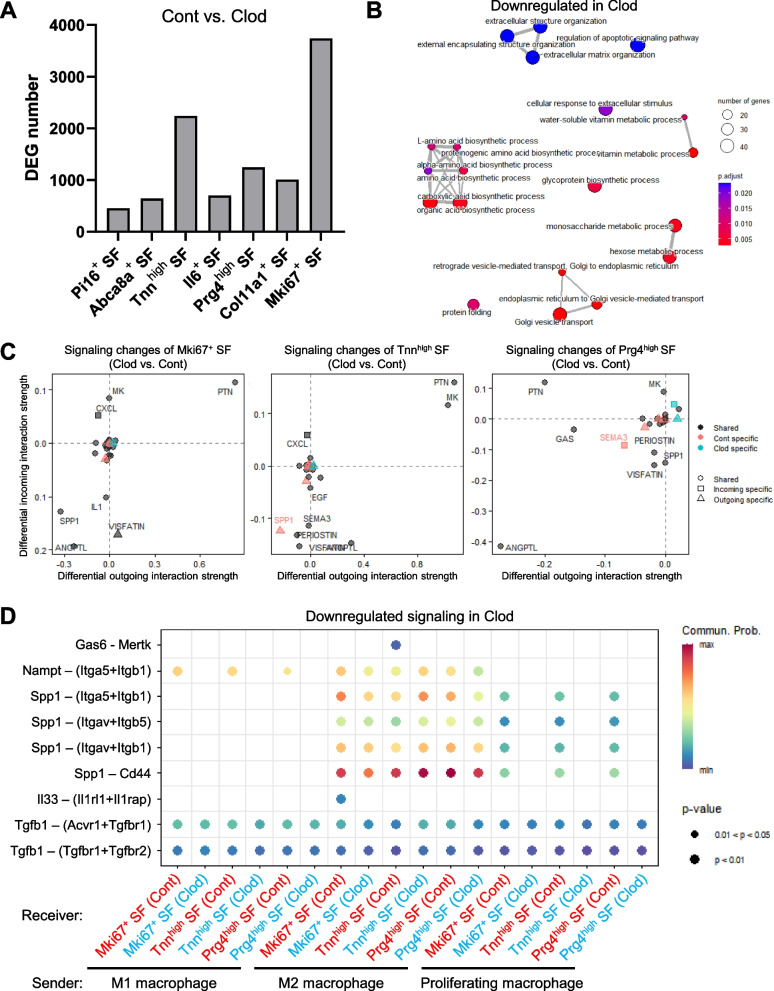
Transcriptome and communication changes of SF subsets. **A** Breakdown of differentially expressed gene (DEG) number between the control and clodronate groups for each SF subset. **B** Enrichment map networks showing gene ontology analysis of DEGs that are downregulated by clodronate treatment in Mki67^+^ SF. **C** Incoming and outgoing signaling changes of Mki67^+^ SF, Tnn^high^ SF, and Prg4^high^ SF. **D** Downregulated signaling received by Mki67^+^ SF, Tnn^high^ SF, and Prg4^high^ SF from macrophages

Examination of the transcriptomic changes in SF subsets between the control and clodronate groups revealed that clodronate treatment resulted in the greatest number of DEGs occurring in Mki67^+^ SF, followed by Tnn^high^ SF and Prg4^high^ SF (Fig. 9A). GO analysis of genes downregulated by clodronate treatment in Mki67^+^ SF identified terms involved in ECM organization, amino acid biosynthesis, and vitamin metabolic process (Fig. 9B). The changes in cartilage ECM production by SFs were confirmed by performing safranin O staining of the synovium (Supplementary Fig. 6). In the control group, the SFs in the superficial layer were slightly stained with safranin O, whereas the clodronate group showed few positively stained SFs.

We then used CellChat [28] to evaluate the changes in cell–cell communication, and we drew the total signaling networks among all cell types as a circle plot (Supplementary Fig. 7A). Both the number of inferred interactions and the interaction strength were greater in the control group than in the clodronate group (Supplementary Fig. 7B). We also identified the signaling networks contributing to dynamic transcriptome changes in Mki67^+^ SF, Tnn^high^ SF, and Prg4^high^ SF by estimating the differential outgoing and incoming interaction strengths due to clodronate treatment (Fig. 9C). For these three SF subsets, incoming signals, such as VISFATIN (i.e., Nampt), SPP1, and ANGPTL, were weaker in the Mki67^+^ SF, Tnn^high^ SF, and Prg4^high^ SF in the clodronate group than in the control group. The outgoing signals from SFs indicated a downregulation of SPP1 in Mki67^+^ SF and Tnn^high^ SF.

We further clarified the contribution of macrophages to these signaling changes by evaluating the changes in signaling from macrophages to Mki67^+^ SF, Tnn^high^ SF, and Prg4^high^ SF (Fig. 9D and Supplementary Fig. 8A). Nampt–(Itga5 + Itgb1) signaling from M1 and M2 macrophages toward Mki67^+^ SF was greatly downregulated by the clodronate treatment. Spp1 signals, such as Spp1–(Itga5 + Itgb1) and Spp1–Cd44, from M2 and proliferating macrophages toward Mki67^+^ SF were also weakened.

**Fig. 10:**
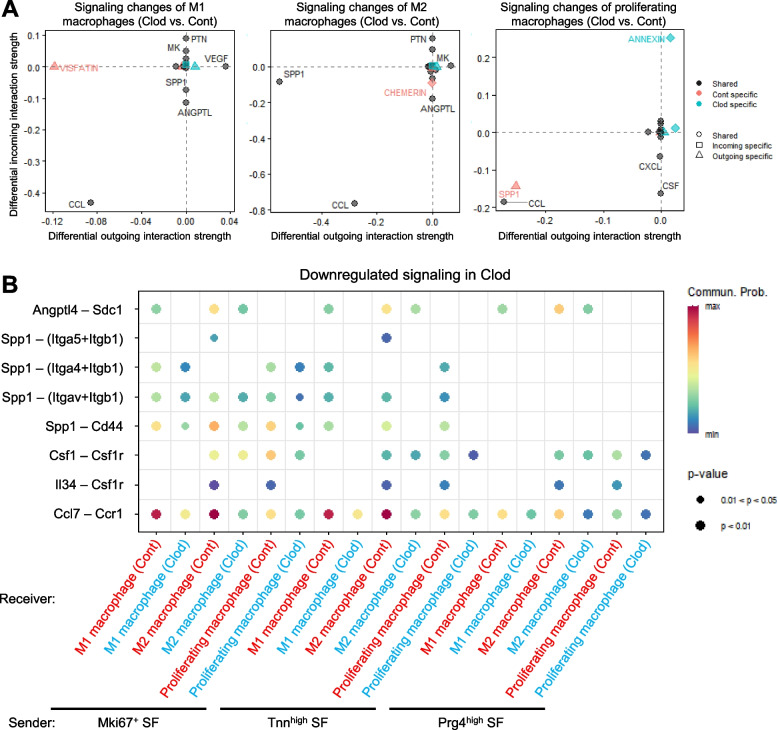
Communication changes in macrophages. **A** Incoming and outgoing signaling changes of each macrophage type. **B** Downregulated signaling received by each macrophage from Mki67^+^ SF, Tnn^high^ SF, and Prg4^high^ SF

We also investigated the intercellular communication occurring between macrophages and other cells (Fig. 10A). The CCL signals received by all three types of macrophages from other cells were attenuated in the clodronate group. At the same time, the Visfatin (Nampt) signal released by M1 macrophages and the SPP1 signal released by M2 and proliferating macrophages were decreased. We also estimated the changes in the signals received by macrophages from Mki67^+^ SF, Tnn^high^ SF, and Prg4^high^ SF (Fig. 10B and Supplementary Fig. 8B). The Ccl7–Ccr1 signaling from Mki67^+^ SF and Tnn^high^ SF to all macrophages showed a relatively higher communication probability than other signals in the control group, but these signals were markedly downregulated in the clodronate group. Spp1 signaling showed a similar trend, although its probability of communication was lower than that of Ccl7–Ccr1. On the other hand, the Csf1–Csfr1 signal from Mki67^+^ SF to proliferating macrophages was reduced in the clodronate group.

### Spp1 and Nampt stimulated the proliferation and/or chondrogenesis of synovial MSCs

**Fig. 11:**
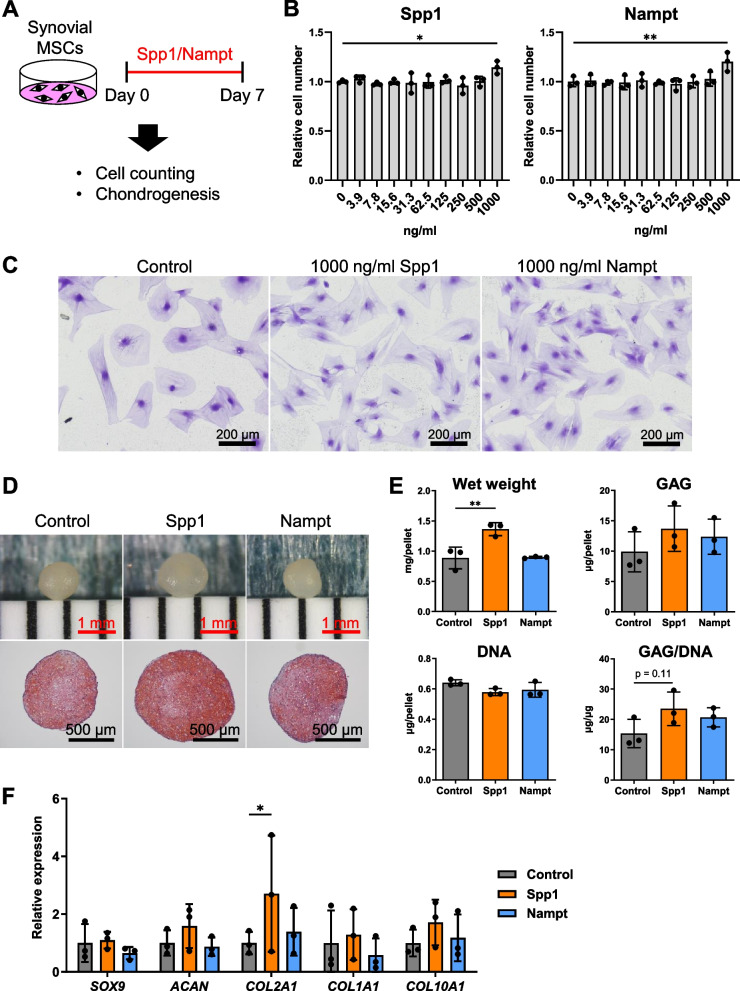
Proliferative and chondrogenic abilities of synovial MSCs treated with Spp1 and Nampt. **A** Experimental scheme. Rat synovial MSCs were treated with recombinant human Spp1 and Nampt for 7 days, followed by cell counting and chondrogenic assays. **B** Relative cell numbers on day 7. Data are presented as the mean ± SD of three replicate wells. **C** Representative images of cells stained with crystal violet. **D** Representative images of gross appearance and safranin O staining of chondrogenic pellets. **E** Pellet wet weight and quantification of glycosaminoglycan (GAG) and DNA. The GAG content was standardized to DNA content (GAG/DNA). Data are presented as the mean ± SD of three replicate pellets. **F** Quantification of chondrogenic genes. Data are presented as the mean ± SD of three replicate pellets. *p < 0.05

Our cell–cell communication analysis revealed that macrophage-derived Spp1 and Nampt signals were downregulated in the clodronate group. Therefore, we investigated the effects of these signals on the proliferation of rat synovial MSCs (Fig. 11A). Spp1 and Nampt treatment at 1000 ng/mL significantly increased cell numbers and the cells assumed a smaller and more spindle-like morphology (Fig. 11 B and C). A subsequent chondrogenic assay using cells treated with 1000 ng/mL Spp1 and Nampt yielded larger chondrogenic pellets that were stained more intensely with safranin O than pellets from the untreated control group (Fig. 11D). Spp1 treatment increased the pellet wet weight and the GAG/DNA ratio compared with the untreated control pellets (Fig. 11E). By contrast, Nampt treatment caused no apparent changes in these features. A subsequent qPCR analysis showed significantly increased *COL2A1* expression in the Spp1 group, but the expression of other chondrogenic genes was comparable in all groups (Fig. 11F).

## Discussion

Our immunohistochemical investigation of the effect of clodronate liposomes in normal rat knees confirmed a marked decrease in the CD68-positive area, indicating that clodronate liposomes effectively eliminated macrophages. In this study, 0.1 mg clodronate was administered twice weekly to the rat knees. Previous reports have indicated that the injection of 0.05 and 0.145 mg clodronate once a week was able to deplete macrophages in mice and rabbits, respectively [19, 20]. Since rats are intermediate in body size to mice and rabbits, a 0.1 mg dose was deemed appropriate.

Carrageenan-induced synovitis was significantly suppressed in the clodronate group. The expression of CD68, CD80, and CD206 was also reduced in the clodronate group, indicating that clodronate was apparently able to deplete macrophages, regardless of their type. Previous studies have reported that the administration of clodronate suppressed synovitis [19, 29, 30], which is consistent with the present results.

The development and promotion of synovitis are directed by macrophages [31]. In the present study, the positive areas for the MSC markers CD44 and CD271 were decreased in the clodronate group, but the CD73-positive area did not change. Kurth et al. [5] reported that MSCs residing in the lining layer express CD44 and CD271, whereas MSCs in the sublining perivascular region express CD271 and CD73. Therefore, our results suggested that the injection of clodronate primarily decreased the number of MSCs in the lining layer. This is probably because the intra-articular injection of clodronate liposomes cannot completely deplete macrophages recruited from blood vessels [15]. Nevertheless, our data confirmed the existence of intercellular communication between macrophages and synovial MSCs.

Our flow cytometry results showed a decreased proportion of macrophages and MSC marker-positive cells in the clodronate group, but the difference was not as large as the difference estimated by the immunostaining results, which represent the positive area (i.e., the number of positive cells). These findings were reasonable, considering that the overall cell number was increased in the control group due to synovitis. Although we observed a shift toward a higher fluorescent intensity of CD206 in the clodronate group, no obvious differences were found in the percentages of CD80 and CD206 marker-positive cells between the two groups. Bailey et al. previously reported that clodronate liposomes increased the M1-to-M2 ratio in a mouse fracture-induced arthritis model [32]. The results of the present study indicated that clodronate clearly decreased the number of macrophages but did not alter macrophage polarity.

The number of colony-forming cells per 1000 synovial cells decreased in the clodronate group. Self-renewal capacity is one of the most important biological functions that characterize synovial MSCs [33]. The number of colony-forming cells per gram of synovium was also decreased by clodronate injection. Together with the results of immunostaining and flow cytometry, the results supported the notion that macrophage depletion caused significant decreases in the proportion and total number of synovial MSCs. These findings provide evidence for active intercellular communication between macrophages and synovial MSCs during synovial inflammation.

We also compared the properties of synovial MSCs cultured from the synovium of the control and clodronate groups, but we found no differences in proliferative ability and surface antigen expressions between them. According to previous studies, in vitro culture can alter the expression of surface antigens of synovial MSCs or can result in the expression of MSC markers in SFs [34]. In our study, the pellet weight and GAG and DNA content per pellet were increased in the clodronate group, but the GAG/DNA ratio was comparable in both groups, indicating that the number of cells in the pellets was larger in the clodronate group, but their chondrogenic potential was the same. Previous reports have indicated that cells without chondrogenic potential are selectively shed during chondrogenic pellet culture [35]. Thus, cultured synovial MSCs from clodronate-injected knees may contain more cells with chondrogenic potential.

The apparent close relationship between macrophages and synovial MSCs led us to perform a confirmatory scRNA-seq experiment. We first examined the cluster to which the synovial MSCs belonged. Among the seven SF subsets identified, we found that the Mki67^+^ SF subset was decreased in the clodronate group. MSCs are normally quiescent; however, when inflammation or injury occurs, they actively proliferate [5]. Our pseudotime trajectory analyses also revealed that this subset is the starting point of the SF lineage. Based on these findings, we considered that the Mki67^+^ SF subset probably corresponded to synovial MSCs. This possibility was further supported by the reduced number of colony-forming cells observed in the clodronate group in the colony formation assay. Consistent with our results, Collins et al. have reported that the Mki67^+^ SF subset appearing after the joint injury was the developmental origin of other SFs in mice [22].

In the clodronate group, the proportion of Col11a1^+^ SF was higher than in the control group. Col11a1 is a marker used to characterize chondrocytes [36]; therefore, Col11a1^+^ SFs are chondrocyte lineage–differentiated cells [22]. Synovial MSCs cultured from clodronate synovium formed larger cartilage pellets in vitro, but showed no difference in the GAG/DNA ratio, possibly because of the higher percentage of Col11a1^+^ SF to total SF. Although Col11a1^+^ SFs may contribute to cartilage regeneration, a previous study by Roelofs et al. demonstrated that the chondrocyte lineage cells in the synovium supplied chondrocytes that formed osteophytes [37]. Thus, chondrocyte-lineage SFs can have both beneficial and detrimental effects in knee joints.

The number of DEG between the control and clodronate groups was greatest for the Mki67^+^ SF, followed by Tnn^high^ SF and Prg4^high^ SF. The expression level of Prg4 and the results of the pseudotime trajectory analyses showed that the Prg4^high^ SF subset was a lining SF subset and that the Tnn^high^ SF subset was an intermediate subset located midway between the Mki67^+^ SF and Prg4^high^ SF. As suggested by the immunohistochemistry results, the clodronate liposome treatment predominantly affected the transcriptomes of cells with a synovial lining lineage. Enrichment analysis of the DEGs downregulated by clodronate treatment in Mki67^+^ SF identified the terms related to ECM organization and amino acid and vitamin biosynthesis. Consistent with the enrichment analysis results, the safranin O staining was less intense in the synovium of the clodronate group than in the control synovium, suggesting no stimulation of GAG synthesis. These findings indicated that macrophage depletion leads to a decrease in the anabolic pathways of ECM and amino acid metabolism in synovial MSCs.

**Fig. 12:**
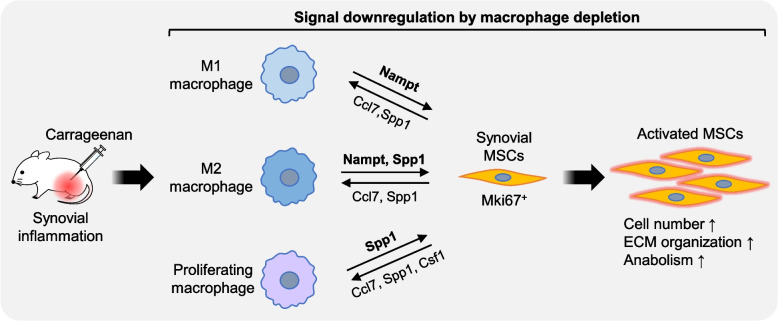
Graphical abstract. Synovial inflammation induced by carrageenan injection causes macrophages to release Nampt and Spp1 to Mki67^+^ synovial mesenchymal stem cells (MSCs). The synovial MSCs release Ccl7, Spp1, and Csf1 to macrophages. As a result, synovial MSCs increase in number and show activation of extracellular matrix organization and anabolic pathways of metabolism. Macrophage depletion by clodronate liposomes downregulated these signals, leading to a lack of synovial MSC activation

We also used CellChat to estimate the changes in ligand–receptor interactions between synovial MSCs and macrophages. The major incoming signals downregulated by clodronate in Mki67^+^ SF included Nampt and Spp1, which were consistent with the signals whose release was suppressed by clodronate in each macrophage type. The Nampt gene codes for nicotinamide phosphoribosyltransferase, also known as visfatin. According to previous studies using zebrafish, Nampt secreted from macrophages after muscle injury acts as a direct inducer of the proliferation and activation of muscle stem cells to promote the regenerative process [17]. Spp1, also known as secretory phosphoprotein 1 or osteopontin, is one of the main osteogenic inducers in MSCs [38]. However, Spp1 has been reported to alleviate the progression of osteoarthritis via Cd44 when injected into knee joints [39]. The Spp1–Cd44 signal is also important for the rejuvenation of adipose MSCs [40]. Although the in vivo potential for enhancement of osteogenesis of synovial MSCs by Spp1 remains unclear, our results suggest a beneficial role of Spp1 signaling in triggering the activation of synovial MSCs. Inflammation and tissue injury are reported to enhance the secretion of Spp1 and Nampt from macrophages [17, 41]. In the present study, clodronate administration greatly suppressed synovitis, possibly through a reduction in Spp1 and Nampt expression in macrophages. In vitro experiments revealed that Spp1 and Nampt treatment promoted the proliferation and/or GAG production of rat synovial MSCs. Although the effects of Nampt and Spp1 signals on synovial MSCs remain to be investigated in vivo, our data support the idea that these signals from macrophages activate synovial MSCs to induce cell proliferation and metabolic changes (Fig. 12).

Other signals, such as Ccl7, Spp1, and Csf1, which were received by macrophages, were downregulated in the clodronate group. Differential expression analysis identified these signals as a form of dysfunctional signaling between each macrophage type and Mki67^+^ SF in the clodronate group. Ccl7 is known to promote macrophage chemotaxis and regulate inflammatory events [42]. Xie et al. reported that CCL7 promoted the M1 polarization of macrophages [43], which may also be involved in the secretory phenotypic changes in macrophages. Spp1 secreted from SFs in a collagen-induced arthritis model is known to promote osteoclast formation via PI3K/AKT signaling [44]. For proliferating macrophages, the Csf1–Csfr1 signaling was downregulated by treatment with clodronate liposomes. Csf1, also known as macrophage colony-stimulating factor, is involved in the proliferation, survival, and differentiation of macrophages [45]. Similar to our results, Knights et al. found SF-derived Csf1 signaling to function as a major crosstalk axis of macrophages activated by injury [46]. Collectively, synovial MSCs are considered to regulate their own activation by regulating their proliferation, polarity, and secretion via Ccl7, Spp1, and Csf1 signaling (Fig. 12).

Understanding the interplay between synovial MSCs and macrophages offers potential therapeutic avenues for the treatment of joint diseases. This study demonstrated the importance of macrophage-derived signals in the activation of synovial MSCs, which are key players in endogenous tissue regeneration. Modulating macrophage activity and/or reproducing their signals may enhance beneficial interactions between synovial MSCs and macrophages, resulting in optimal synovial MSC activation and tissue regeneration [47]. However, we recognize two limitations of our study. One is that the intra-articular injection of clodronate liposomes did not achieve complete macrophage depletion. As macrophages are also supplied via the bloodstream [15], the systemic administration of clodronate liposomes should be considered for the complete elimination of macrophages. Another limitation is that the communicational changes between synovial MSCs and macrophages that we identified here are just estimates. Although we confirmed the in vitro effects of Spp1 and Nampt, we need to confirm the significance of our findings through in vivo experiments to better understand each signaling contribution.

## Conclusions

The present study investigated the changes in the number and function of synovial MSCs when macrophages were depleted by clodronate liposome treatment of acutely inflamed knees. The clodronate liposome treatment suppressed synovitis and decreased synovial MSC numbers and proportions. Synovial MSCs cultured from synovia removed from control and clodronate-treated rats showed comparable in vitro surface antigen expression, proliferative capacity, and chondrogenic potential. Our scRNA-seq analysis demonstrated decreased numbers of Mki67^+^ SF corresponding to synovial MSCs and downregulated expression of genes related to ECM organization and anabolism in the clodronate group. Our cell–cell communication analysis confirmed that macrophage depletion suppressed Nampt and Spp1 signaling from macrophages to Mki67^+^ SF and suppressed Ccl7, Spp1, and Csf1 signaling from Mki67^+^ SF to macrophages. Spp1 and Nampt treatment promoted the in vitro proliferation and/or chondrogenesis of synovial MSCs. Our results verified that macrophage-derived signals during inflammation are important for the activation of synovial MSCs and have provided clues for the development of novel therapeutic strategies that can stimulate tissue regeneration by endogenous synovial MSCs.

## Supplementary Information

Additional file 1: Supplementary Fig. 1. CD68 immunostaining of the synovium of intact knees. A Representative images of CD68. B Quantification of the positive areas. Data are presented as the mean ± SD of six knees. ****p < 0.0005. Supplementary Fig. 2. Clustering results of single-cell RNA sequencing before cell type annotation. **A** Uniform manifold approximation and projection (UMAP) plot showing 20 distinct clusters for integrated data. **B** UMAP plot for each condition. Supplementary Fig. 3. Expression patterns of marker genes used for the cell type annotation. Supplementary Fig. 4. Proportional breakdown of each subset. A Proportion of the total cell population represented by each cell type. B Proportion of the total macrophage population represented by each macrophage type. Supplementary Fig. 5. Heatmap drawn using the top 10 DEGs between seven distinct SF subsets. Supplementary Fig. 6. Representative images of safranin O staining of synovium from each treatment group. Supplementary Fig. 7. Signaling networks among all cell types. **A** Circle plot showing total signaling networks among all cell types in each condition. **B** Number of inferred interactions and interaction strength in each condition. Supplementary Fig. 8. Signaling network between Mki67^+^ SF, Tnn^high^ SF, and Prg4^high^ SF and macrophages. **A** *Chord* diagram showing signaling from macrophages to Mki67^+^ SF, Tnn^high^ SF, and Prg4^high^ SF in each condition. **B** *Chord* diagram showing signaling from Mki67^+^ SF, Tnn^high^ SF, and Prg4^high^ SF to macrophages in each condition.

Additional file 2: Supplementary table. List of all differentially expressed genes between the control and clodronate groups for each SF subset.

## Data Availability

The datasets generated and analyzed during the current study are available from the corresponding author on reasonable request. The scRNA-seq datasets were deposited in the National Center for Biotechnology Information’s Gene Expression Omnibus with accession number GSE269120.

## Abbreviations

SFs: Synovial fibroblasts
MSCs: Mesenchymal stem cells
scRNA-seq: Single-cell RNA sequencing
EDTA: Ethylenediaminetetraacetic acid
HE: Hematoxylin/eosin
TBS-T: Tris-buffered saline containing 0.1% Tween-20
α-MEM: α-Modified essential medium
FBS: Fetal bovine serum
qPCR: Quantitative real-time PCR
GAG: Glycosaminoglycan
