# Supplementary material for: Macrophage depletion in inflamed rat knees prevents the activation of synovial mesenchymal stem cells by weakening Nampt and Spp1 signaling
Source: Inflamm Regen. 2024 Nov 20;44:47. doi: 10.1186/s41232-024-00361-2 (PMC11577658; doi:10.1186/s41232-024-00361-2)

Supplementary Fig.1

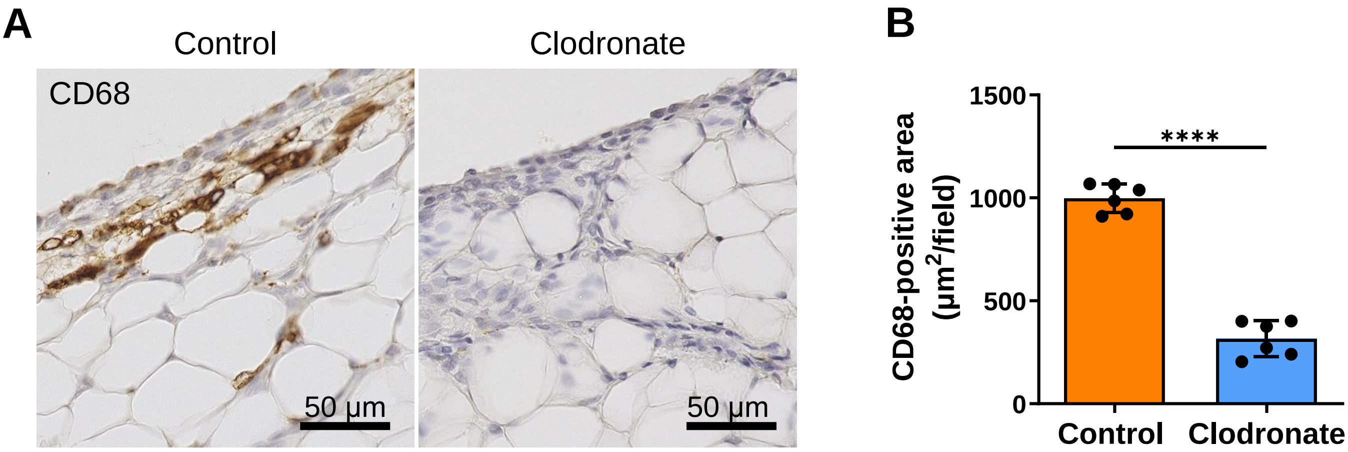

Supplementary Fig.2

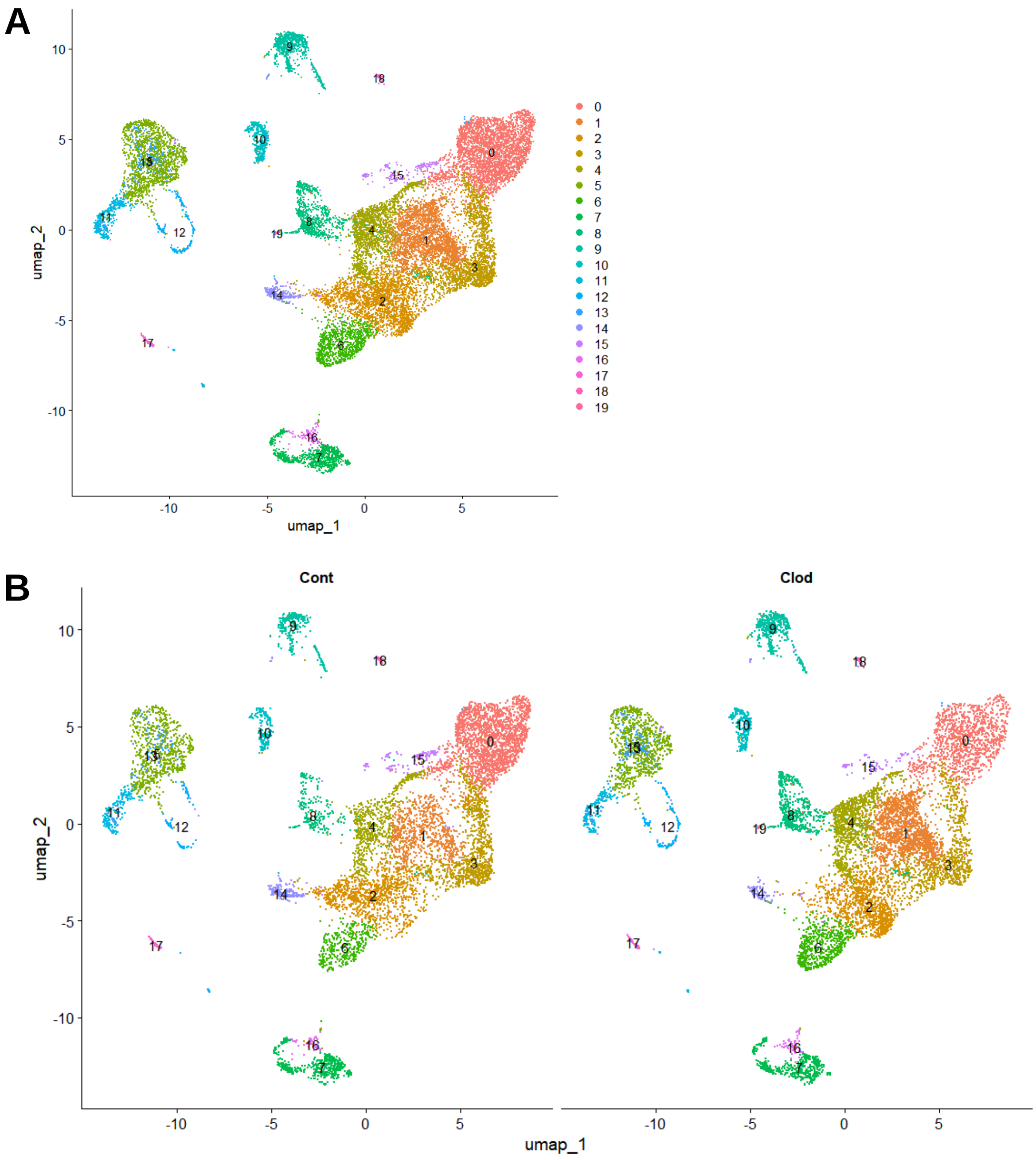

Supplementary Fig.3

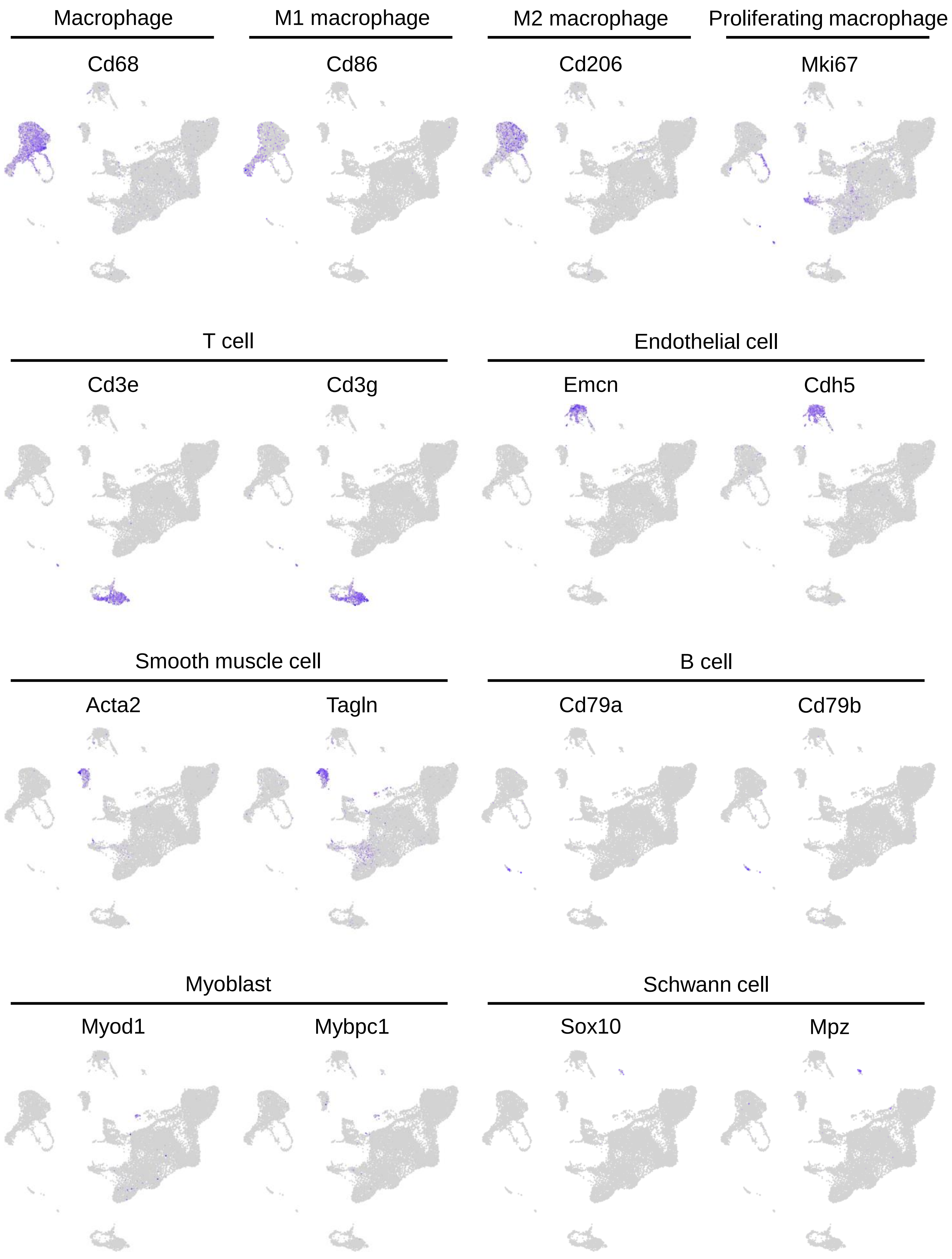

Supplementary Fig.4

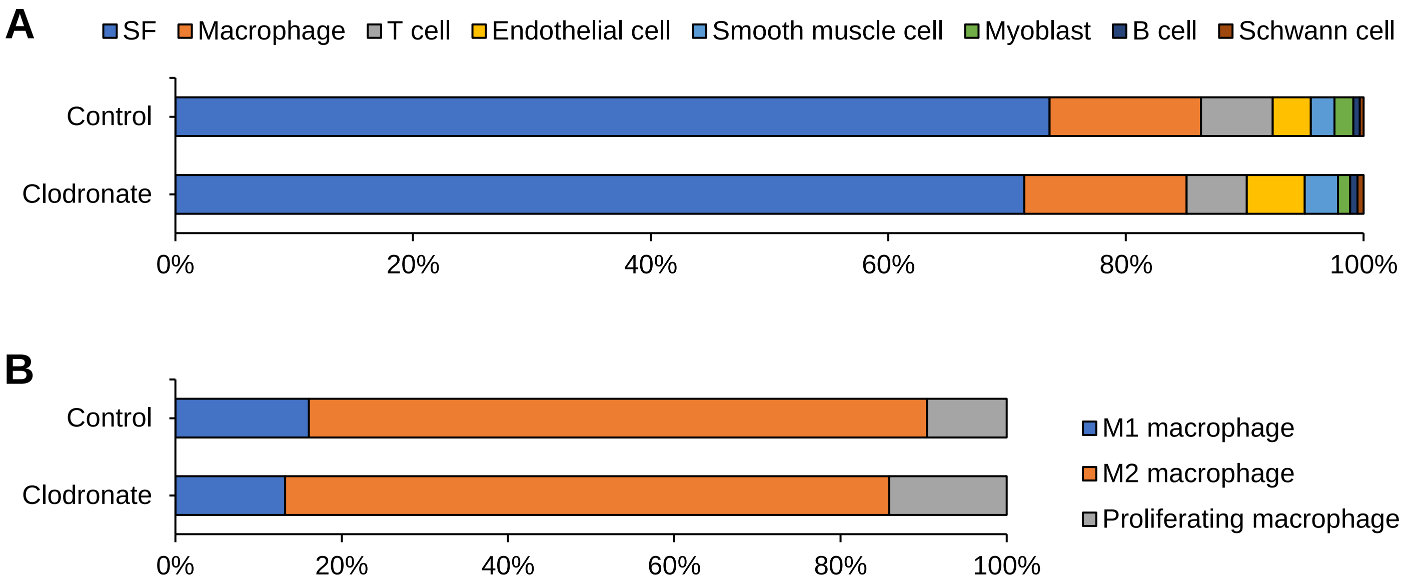

Supplementary Fig.5

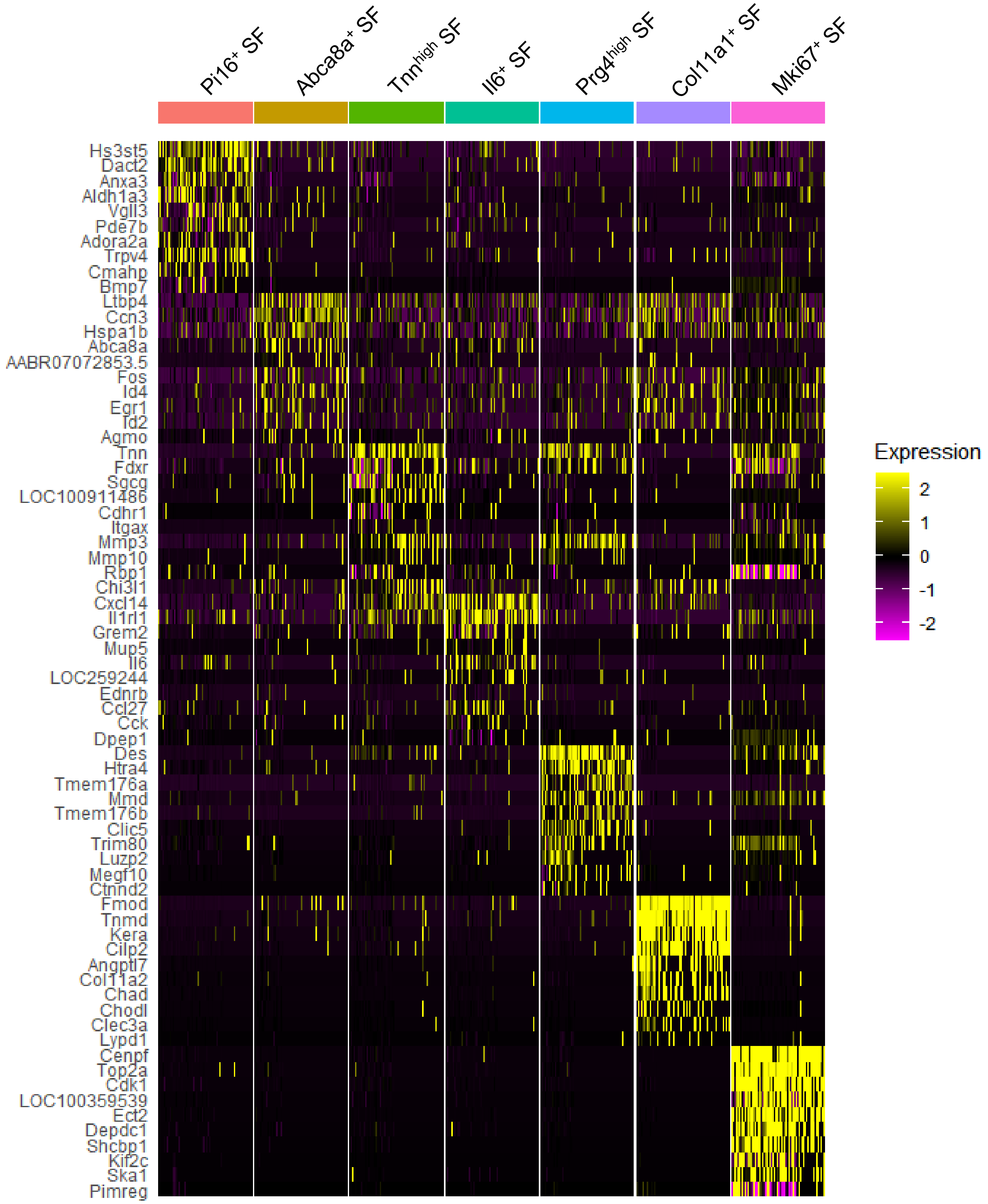

Supplementary Fig.6

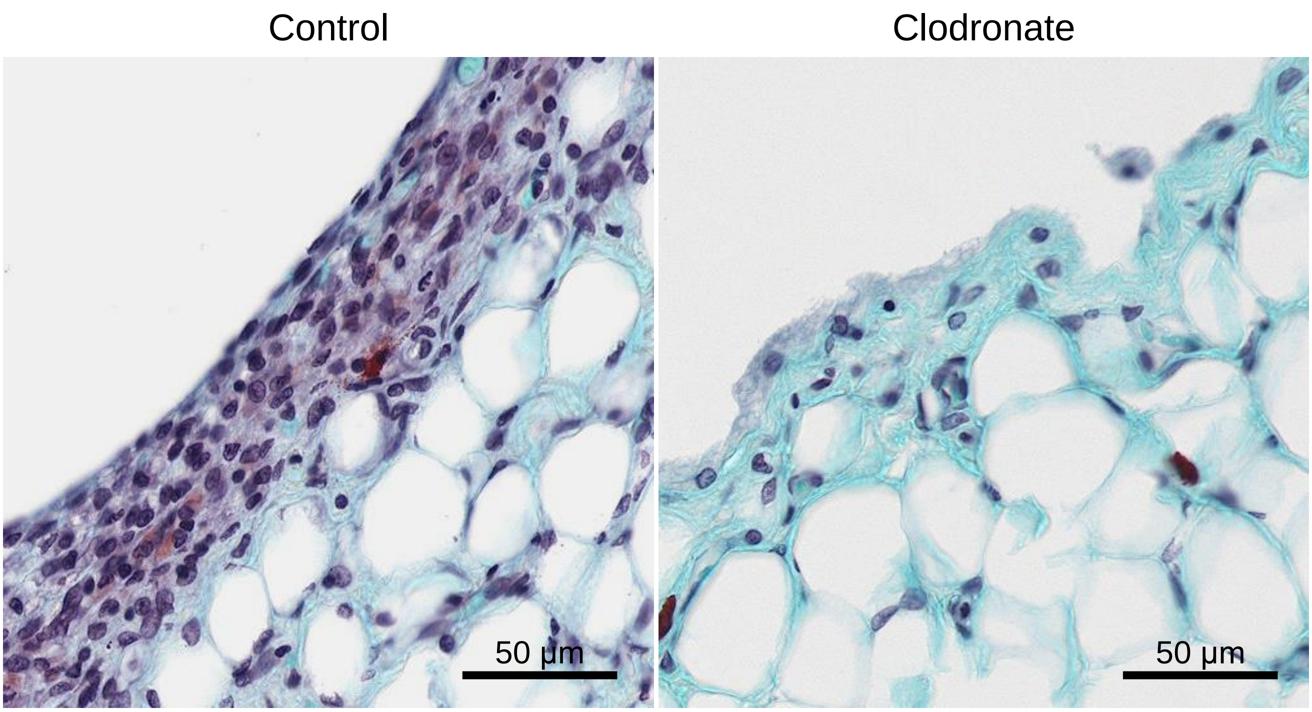

Supplementary Fig.7

A

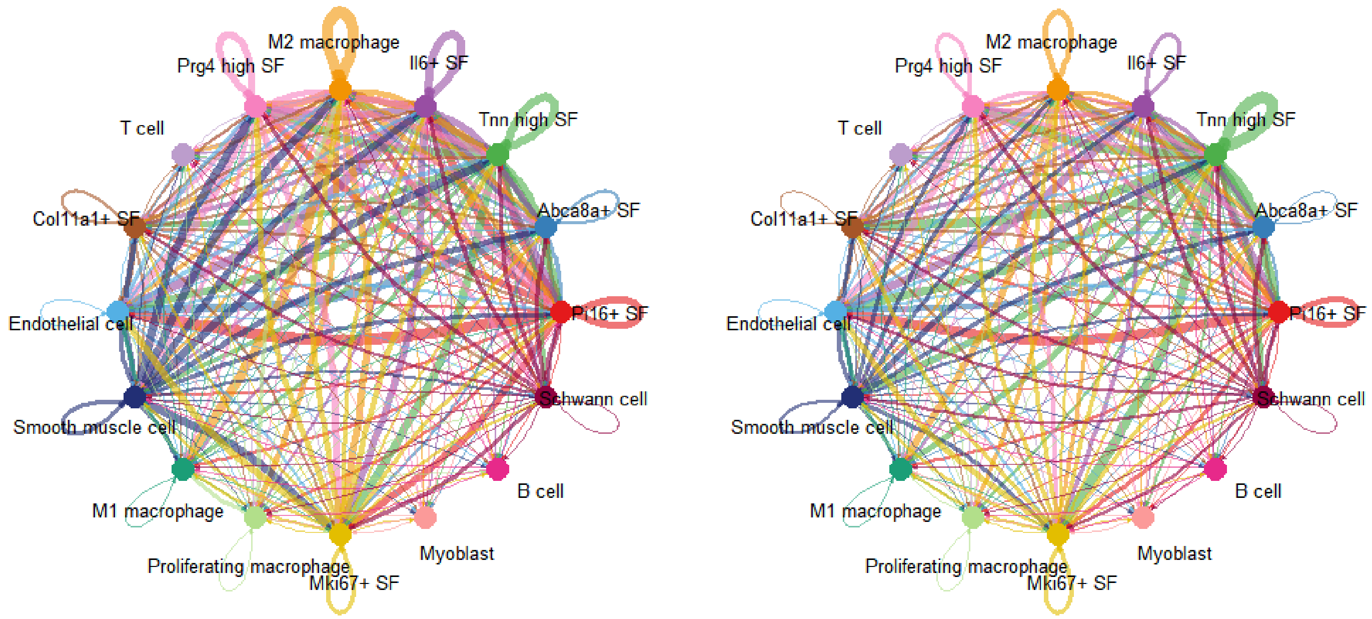

B

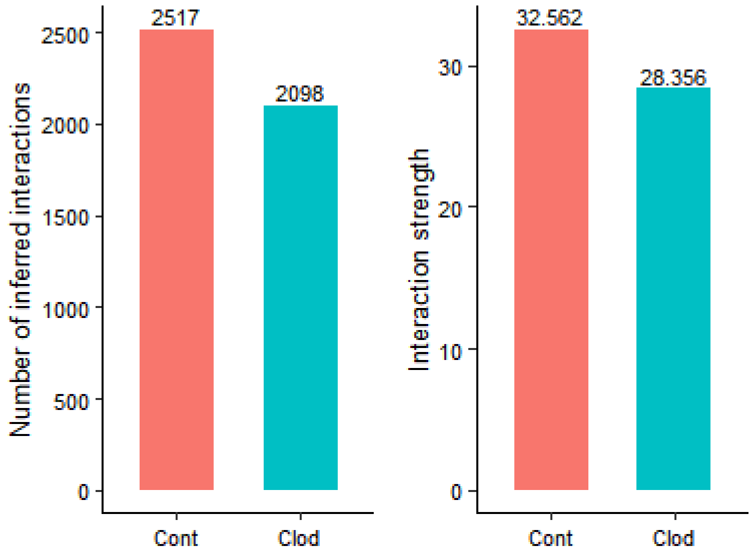

Supplementary Fig.8

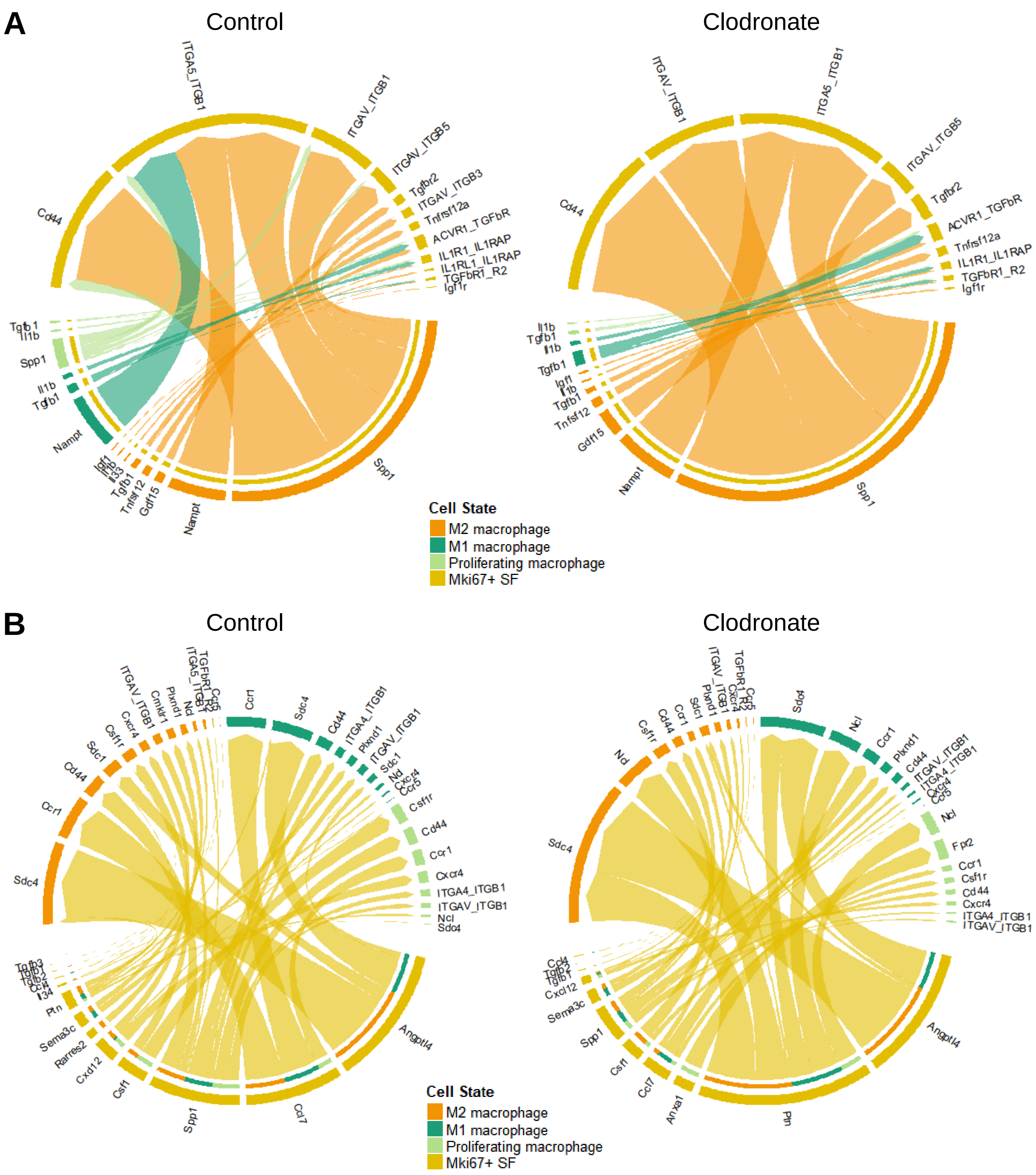

Supplement: Supplementary file 1 — Additional file 1: Supplementary Fig. 1. CD68 immunostaining of the synovium of intact knees. A Representative images of CD68. B Quantification of the positive areas. Data are presented as the mean ± SD of six knees. ****p < 0.0005. Supplementary Fig. 2. Clustering results of single-cell RNA sequencing before cell type annotation. A Uniform manifold approximation and projection (UMAP) plot showing 20 distinct clusters for integrated data. B UMAP plot for each condition. Supplementary Fig. 3. Expression patterns of marker genes used for the cell type annotation. Supplementary Fig. 4. Proportional breakdown of each subset. A Proportion of the total cell population represented by each cell type. B Proportion of the total macrophage population represented by each macrophage type. Supplementary Fig. 5. Heatmap drawn using the top 10 DEGs between seven distinct SF subsets. Supplementary Fig. 6. Representative images of safranin O staining of synovium from each treatment group. Supplementary Fig. 7. Signaling networks among all cell types. A Circle plot showing total signaling networks among all cell types in each condition. B Number of inferred interactions and interaction strength in each condition. Supplementary Fig. 8. Signaling network between Mki67+ SF, Tnnhigh SF, and Prg4high SF and macrophages. A Chord diagram showing signaling from macrophages to Mki67+ SF, Tnnhigh SF, and Prg4high SF in each condition. B Chord diagram showing signaling from Mki67+ SF, Tnnhigh SF, and Prg4high SF to macrophages in each condition. [file 41232_2024_361_MOESM1_ESM.pdf]
